# Metals and Metal-Nanoparticles in Human Pathologies: From Exposure to Therapy

**DOI:** 10.3390/molecules26216639

**Published:** 2021-11-02

**Authors:** Joanna Izabela Lachowicz, Luigi Isaia Lecca, Federico Meloni, Marcello Campagna

**Affiliations:** Division of Occupational Medicine, Department of Medical Sciences and Public Health, University of Cagliari, 09048 Monserrato, CA, Italy; lachowicz@unica.it (J.I.L.); luigiisaia.lecca@unifi.it (L.I.L.); federicomeloni@hotmail.it (F.M.)

**Keywords:** metal toxicity, metal nanoparticles, toxicity, chelating therapy

## Abstract

An increasing number of pathologies correlates with both toxic and essential metal ions dyshomeostasis. Next to known genetic disorders (e.g., Wilson’s Disease and β-Thalassemia) other pathological states such as neurodegeneration and diabetes are characterized by an imbalance of essential metal ions. Metal ions can enter the human body from the surrounding environment in the form of free metal ions or metal-nanoparticles, and successively translocate to different tissues, where they are accumulated and develop distinct pathologies. There are no characteristic symptoms of metal intoxication, and the exact diagnosis is still difficult. In this review, we present metal-related pathologies with the most common onsets, biomarkers of metal intoxication, and proper techniques of metal qualitative and quantitative analysis. We discuss the possible role of drugs with metal-chelating ability in metal dyshomeostasis, and present recent advances in therapies of metal-related diseases.

## 1. Introduction

Metals have unique chemical-physical properties that cannot be provided by organic compounds. Indeed, redox reactions that transfer electrons in biological processes and provide energy for every single cell are run in the metal-catalytic center (i.e., iron, copper, manganese) of enzymes. Other metals (i.e., calcium, magnesium, sodium) maintain the electron gradient throughout the cell membranes. At least 10 metals are defined as essential for humans and are indispensable in biochemical processes; nevertheless, 46 metals (including radionucleotides) are used in their pure form or as metallodrugs in pharmacotherapies, diagnosis, and theragnosis [1]. 

Humans are exposed to a plethora of nanoscale particles present in the environment. Nanoparticles (NPs)—particles whose diameter is lower than 100 nanometers (10^−9^ m), ranging from 1 to 100 nm [2]—have different shapes and compositions, which define their unique properties and interactions with human cells. Environmental NPs are by-products of combustion processes (e.g., coal, petroleum, and wood burning), automobile exhaust, aerosols from atmospheric processes, and the activity of volcanoes [3]. In this way, environmental NPs have different chemical compositions and forms. Conversely, industrial NPs have a defined chemical composition and homogeneous shape. Industrial nanomaterials (NMs)—often composed of heavy metals (e.g., nickel, cadmium, manganese, zinc, titanium, gold, antimony, silicon, and their metal oxides), carbon, and others can be engineered or incidentally released in the environment [4]. Among over 1800 consumer products containing engineered nanoparticles, most of them are metal-NPs [5,6]. Metal NPs can enter the human body mainly through the respiratory, dermal, gastrointestinal, circulatory, immunological, and neurological tracts [7]. Once absorbed, NPs can exert their toxic effects immediately or after translocation to the target organ. Clinically, it is difficult to distinguish between the intoxication of metal-NPs and metal ions, and it is difficult to establish whether metal-NPs were formed in vitro or in vivo as metal aggregates.

**Scheme 1:**
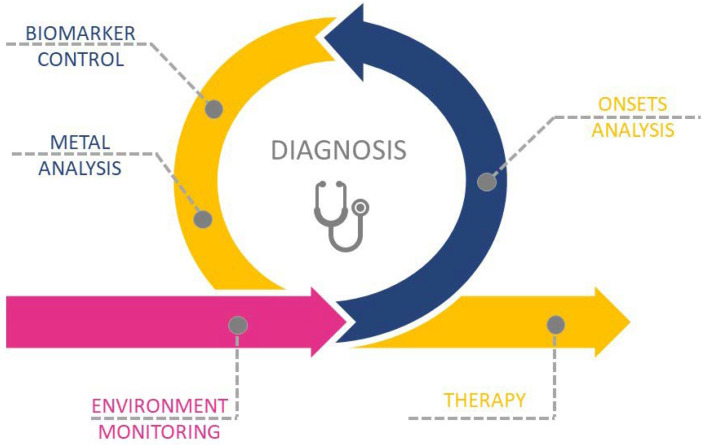
Schematic representation of the multi-step process in metal-related diseases diagnosis and therapy.

The aim of this review is to present recent advances in metal-related pathologies and clinical bias in the recognition of metal or metal-NPs intoxication. We present the metal-related pathologies and discuss the importance and way of environmental monitoring, which is a first step in the recognition and assignment of metal-related pathologies. We present different steps of correct diagnosis (Scheme 1): from onsets analysis, biomarker control, and metal qualification and quantification; presenting the challenges in the fast and unequivocal recognition of metal toxicity. We pay particular attention to the metal accumulation sites, the possible translocation between tissues, and the potential role of drugs in etiology and enhancement of metal toxicity and dyshomeostasis. Finally, we present the recent advances in the therapy of metal-related diseases. 

## 2. Pathologies Correlated with Metal Dyshomeostasis

Metal ions play a structural (e.g., calcium in bones) and regulatory (e.g., iron in redox processes) role in the human body. The proper functioning of metal-related processes is strictly regulated by a series of proteins that store and translocate metal ions. Any unbalance in metal concentration and/or metal-regulating proteins leads to metal dyshomeostasis and the development of severe pathologies. ‘Dose-effect’ correlation is fundamental for each metal-related disease and describes the quantifiable toxic effect in the function of metal concentration (‘all-or-non’ relation if the toxic effect is not quantifiable). Non-essential metal ions always lead to undesired processes, which effects are directly correlated with the metal concentration. Unphysiologically low or high concentrations of essential metal ions also lead to the development of pathological states.

The etiology of metal-related pathologies can be genetic, when the metal-regulating proteins are unbalanced due to genetic disorder, or sudden when the metal ion concentration is increased by an uncontrolled absorption. There is also a group of pathologies correlated with metal dyshomeostasis, for which the etiology is still unknown.

### 2.1. Genetic Disorders

Metal-related pathologies due to genetic disorders occur in the absence of environmental metal exposure and are related to insufficiency or overload of essential metal ions. The genetic dysfunction of metal-related proteins leads to ‘toxic-loss-of function’ in the destination locus and ‘toxic-gain-of-function’ due to metal overload in affected tissue(s).

Among 10 essential metal ions, iron-genetic disorders are the most diffused and are caused by the mutation of different genes; leading to distinct pathologies (Table 1). Mutations in iron-transport and storage proteins (Table 1) cause the accumulation of iron ions and their precipitates, leading to acute inflammation of the tissues. Manganese and copper are other two essential metal ions, which are overloaded in the absence of their accompanying proteins, and lead to inflammation of the liver and nervous system. Of note, regular biochemical pathways of each metal are correlated with other metals, thus iron, copper, and manganese dyshomeostasis lead to unbalance of other essential metal ions, particularly zinc [8,9], and could also increase susceptibility to metal (both toxic and essential) intoxication.

Genetic disorders with systemic metal accumulation or insufficiency are widely described in scientific and clinical literature [29,30], thus are much easier to diagnose with respect to other metal-related pathologies. Each genetic polymorphism can be screened and properly associated with the disease, while Magnetic Resonance Imagining (MRI) non-invasive analysis help to diagnose and let to monitor the metal overload in target tissues.

In the last century, intensive studies in the field of pharmacological chelation therapy and metal supplementation in the treatment of metal-overload and metal-insufficiency genetic disorders deliver on the market number of therapies (Table 1) that could be potentially used in the treatment of metal dyshomeostasis unrelated to genetic polymorphism.

### 2.2. Acute and Chronic Metal Intoxication

Military actions and intensive industrial production are correlated with metal intoxications. The Second World War and the successive fast economic and industrial growth entered the medical history with record numbers of pathologies and deaths due to uncontrolled contact with enormous quantities of toxic metals.

The end of the Cold War in the 90s together with growing medical, scientific, and social awareness of the metal hazards permits to control tightly metals in the military and everyday products industry. As a result, the overall number of pathologies caused by metals has significantly decreased compared to the second half of the 20th century. However, there are still cases of intoxications with metals, often coming from the surrounding environment (e.g., cadmium) and food products (e.g., arsenic). Particular attention should be focused on intoxications with metals, which recently entered the industry due to the development of new technologies (e.g., thallium), often in the form of metallic nanoparticles (e.g., nickel). We should also pay attention to the medical literature over the last years, which records a growing number of case studies with metals originating as drugs (e.g., lithium) or their formulations (e.g., aluminum).

Thallium salts are now considered to be amongst the most toxic compounds known. Nevertheless, the high-thallium permeability of the nervous system is used in medical diagnostics of brain tumors and brain trauma evaluation [31]. Even if the nervous system is a very important target of this element, most of the thallium toxicity studies have been focused on non-neuronal tissues such as the liver, kidney, muscle, and in the cardiovascular system [31]. Thallium is used industrially in small quantities, mainly in electronics, in the production of certain glasses, and crystals and in medical diagnostics. An epidemiological study in an area with high thallium concentrations in soil and garden vegetables centered on a cement plant showed a dose-response relationship between thallium concentration in urine and a number of non-specific subjective symptoms [32].

Lithium is frequently used in the treatment of bipolar disorders, but it is also known to induce electrocardiogram (ECG) alterations and cardiotoxicity including sinus node dysfunction [33,34]. When prescribing lithium, the risk of toxicity remains a concern. As shown by Ott et al. [35], some patients in lithium therapy experienced at least one episode of lithium levels ≥ 1.5 mmol/L (an incidence of 0.01 per patient-year), of whom 34% required intensive care and 13% were treated with hemodialysis. Lithium intoxication seems rare and can be safely managed in most cases, but physicians should screen patients for eventual toxicity.

Arsenic is a metalloid found in drinking water, soil, plants, fish [36], cigarettes [37], and cereals, but the main source of arsenic intoxication is contaminated water. Cadmium is present in traffic-related emissions [38], fertilizers, water [39], cigarettes and various food (grains and vegetables) [40]. Mining activities of zinc and iron extraction, along with battery production are the main industrial activities leading to the increased presence of cadmium in the environment. Both arsenic and cadmium are endocrine disruptors due to the activation of different molecular mechanisms, hence they differ in their initiated molecular events, which later lead to breast cancer and prostate cancer onset [41]. It is well ascertained that cadmium is a carcinogen, while arsenic carcinogenicity is still to ascertain. Of note, arsenic (III) oxide (As_2_O_3_) is used in the treatment of chronic myelogenous leukemia.

Exposure to various trace elements can cause the clinical features observed in Mesoamerican Nephropathy (MeN). Particularly low-dose, chronic environmental exposure to nickel is a possible health risk MeN. Nickel intoxication and resulting systemic and renal effects could explain the clinical signs observed during the early stages of the disease. Moreover, Fischer et al. [42] provided compelling evidence for a role of Ni in the acute renal impairment observed in this MeN high-risk population.

In the light of recent studies, pregnancy as well as some pathologies can increase susceptibility to metal dyshomeostasis and further intoxication. For instance, a positron emission tomography (PET) study with radioactively labeled iron in humans with Wilson Disease patients showed the increased iron uptake by the brain [43]. According to the recent studies by Tomska et al. [44], there is a correlation between zinc, copper, and cadmium concentrations in the human body and the environment in various geographic locations in Poland, which can be of importance for both the proper development and the course of pregnancy. Zinc, copper, and cadmium interact, with each other in the human body and their co-existence, causes a reduction in their levels [45]. Tomska et al. [44] analyzed the zinc:cadmium and zinc:copper molar ratio in the umbilical cord, placenta, and fetal membrane, showing that there are significant differences in the accumulation of cadmium, zinc, and copper, depending on the place of residence of the study participants.

Aluminum as a free metal cation (Al^3+^) is highly biologically reactive, and it is essentially toxic. Aluminum can be found throughout the human body, but its acute toxicity is rare. Nevertheless, little is known about chronic aluminum intoxication. Of note, not all forms of aluminum are toxicologically equivalent and not all routes of exposure are equivalent in their delivery of aluminum to target sites [46]. Despite intensive studies correlating Alzheimer’s disease with chronic aluminum intoxication; or breast cancer with topical application of an aluminum salt; or even autism with immune cascade initiated by an aluminum adjuvant; there is no proof of straightforward relation of aluminum and listed pathologies [46].

### 2.3. Multifactorial Etiology

There are groups of uncurable diseases of undefined etiology-most probably multifactorial, namely diabetes and neurodegeneration. Currently, the only known common feature of these two groups of pathologies is misfolding protein process [47,48]. Protein misfolding occurs under unbalanced pH, temperature, pressure, ionic strength, protein concentration conditions with contemporary dysfunction of the protein degradation system [49]. Overrange conditions can be a result of environmental and/or genetic factors. Metal ions are among environmental factors that significantly influence protein aggregation and are linked to neurodegeneration and diabetes [48,50].

Calcium, iron, copper, manganese, zinc, and selenium are essential for brain physiology, and metal concentration unbalance leads to neurons dysfunction, increases oxidative stress, and DNA damage. Neurons have high metabolic turn-over, resulting in reactive oxygen species (ROS) production in the respiratory chain [51]. Moreover, abundant unsaturated fatty acids in neuronal cell membranes increase the probability of their oxidation resulting in lipid peroxidation [52]. Surprisingly, brain concentrations of low antioxidant enzymes and low molecular weight antioxidants are much lower than in other tissues [53]. For instance, cytosolic glutathione (GSH) content is approximately 50% lower in neurons compared to hepatocytes. In addition, an increase in redox-active metals (e.g., iron, manganese, and copper) in the brain was observed in neurodegenerative pathologies. The total iron in the affected brain area of patients with progressive supranuclear palsy (PSP) (globus pallidus of PSP patients 257 ± 19 ng/mg iron (control 183 ± 22 ng/mg iron); substantia nigra of PSP patients 301 ± 26 ng/mg iron (control 188 ± 22 ng/mg Fe)) was much higher than in the age-matched patients without neurodegenerative pathologies. Moreover, increased concentrations of labile, non-ferritin iron have been detected in Parkinson’s (PD) and Alzheimer’s Disease (AD) (substantia nigra PD patients 534 ± 71 ng/mg H-rich ferritin (control 375 ± 38 ng/mg H-rich ferritin); hippocampus AD patients 29 ± 5 ng/mg H-rich ferritin (control 9 ± 2 ng/mg H-rich ferritin)) [54]. Importantly, iron overload, of an exogenous or endogenous origin, affects neuronal genomic stability. Moreover, increased ROS production in the Fenton reaction leads to apoptotic and ferroptotic neuronal death [54].

Among all human tissues, zinc concentration is the highest in the brain, e.g., exceeding ten times liver and serum [55]. High zinc content is found in hippocampus, amygdala, and dentate gyrus [55]. Moreover, 80–90% of neuronal zinc is tightly bound to metal-binding proteins, while the remaining 10–20% pool is stored in synaptic vesicles of excitatory neurons [56]. Zinc is a catalytic, structural, or regulatory component of various proteins involved in different physiological processes, namely neurotransmission, enzymatic activity, gene regulation, structural preservation, and stabilization of proteins [56]. Both zinc-deficient and zinc-overload are correlated with increased oxidative stress and pathophysiological neuronal changes such as aging-associated diseases [57].

Copper is the third abundant trace element in the brain after iron and zinc, with the highest levels in the substantia nigra, locus coeruleus (both containing catecholaminergic cells), dentate nucleus, basal ganglia, hippocampus, and cerebellum [55]. Microarray studies with RNA samples from brains of AD patients revealed reduced mRNA expression of the copper-dependent enzymes (e.g., superoxide dismutase 1 (SOD1) and antioxidant protein 1) that was associated with a reduced copper concentration in the brain. Copper dyshomeostasis, in the nervous system, leads to pathological conditions associated with neurodegenerative pathologies such as AD. Potential modes of action appear to be among others the induction of oxidative stress and resulting DNA damage. Impairment of plasma copper levels may determine abnormal brain copper levels in AD [58]. In AD patients, serum copper levels were approximately 54% higher than in controls. Moreover, the presence of the epsilon4 allele of the apolipoprotein E (APOE) gene, an important genetic risk factor for AD, is associated with higher serum copper concentrations [59].

The role of copper in promoting Parkinson’s disease has been confirmed in several epidemiological studies. For instance, long-term exposure to copper in the workplace is associated with an increased risk of developing Parkinson’s disease [60]. Moreover, the presence of free copper ions in the brain causes undesired oxidation of dopamine neurotransmitter [61]. Dopamine oxidation can also be mediated by copper ions associated with various ligands or peptides/proteins that are involved in the process of neurodegeneration [62].

Manganese constitutes the enzymatic center of various metalloproteins, such as phosphoenolpyruvate decarboxylase, glutamine synthetase, arginase, pyruvate carboxylase, and manganese-SOD enzymes, which regulates the cellular redox status, mitochondrial function, and neurotransmitter synthesis [63,64]. Human manganese brain concentration increases with age and was correlated with PD [65]. Recent studies indicate a contribution of manganese-induced oxidative stress and genomic instability by means of manganese-induced DNA damage.

Selenium is preferentially transported to the brain at the expense of other tissues to ensure sufficient concentrations under conditions of nutritional deficiency [66]. Selenium as selenocysteine becomes incorporated into selenoproteins, which regulates the antioxidative process in the tissues [67]. Ferroptotic cell death, modulated by p53 and increased concentrations of free Fe, frequently occurs in neurons, which can be counteracted by glutathione peroxidase 4 (GPX4) [68]. A recent study showed that a single dose of selenium (1 μM) delivered into the brain enhances expression of GPX4 and other selenoproteins, and protects neurons from ferroptosis in a hemorrhagic stroke model [69].

Calcium acts as a second intracellular messenger and plays a key role in the regulation of neuronal functions, namely neural growth and differentiation, action potential, and synaptic plasticity [70]. Calcium signaling is involved in neurotransmitter release, synaptic plasticity, gene expression, and other important neuronal functions [71]. The so-called “Calcium hypothesis” was first postulated by Khachaturian in 1989 [72]. According to this hypothesis, the depolarization of aged neurons causes the influx of calcium ions from the extracellular space and excitotoxicity. Other studies demonstrated that neuronal aging is associated with the alteration of neuronal calcium extrusion [73]. Progressive overproduction and accumulation of amyloid-beta (Aβ), the main misfolding protein involved in AD, cause the dysregulation of calcium homeostasis. Aβ disrupts calcium signaling by increasing the influx of ions from the extracellular space and by activating its release from intracellular stores [74].

## 3. Exposure, Diagnosis, and Therapy of Metal-Related Diseases

Metal toxicity lacks specific symptoms, particularly when the exposition is time-long and at low doses. Environment monitoring of workplaces and habitats permits avoiding exposition to metal ions, and if uncontrolled exposure occurs, it leads to indicate population exposure to hazards. Next to monitoring data, some common onsets of metal intoxication are an indication for the analysis of metal-specific biomarkers. Metal intoxication can be confirmed by the specific qualitative and quantitative analysis in the target tissue, and particular attention must be taken to the choice of biological samples and proper analytical techniques. Different drugs, often used in life-long therapy, have metal-binding sites, which lead to the formation of metal complexes, and could be involved in etiology and/or enhancement of metal dyshomeostasis and successive intoxication.

### 3.1. Environmental Monitoring

Metals and metal-NPs are released to the atmosphere (aerosols), as well as to the soil and surface water, and remain in circulation for a prolonged period of time, taken up by biological organisms (particularly metals and metal-NPs with very low solubility or degradability). The released metals and metal-NPs can be bare metals or nanoparticles, functionalized nanoparticles, aggregates, or even particles embedded in a matrix. Some metals and metal-NPs have ecotoxicological hazards, bioaccumulate in the food chain, or are biodegraded [75]. Degradation products released from biodegradable particles can exert short or long-term biological effects [75]. Particular attention should be given to degrading metal-NPs, which release free metal or their ionic forms (aqueous solutions) into the environment.

Nowadays, numerous metal-NPs are used in daily life products. Silver (AgNPs), gold (AuNPs), copper (CuNPs), palladium (PdNPs), titanium dioxide (TiO_2_ NPs), zinc oxide (ZnO NPs), and copper oxide (CuO NPs) are exploited in antimicrobial/antiseptic, medical, catalytic, and electronic processes, due to their metal-related chemical-physical properties [76,77,78,79,80]. The morphology and properties of metal-NPs can be adjusted under different conditions of pH, oxygen concentration, and the presence of radical species, organic matter, and other metal and metal oxide particles/nanoparticles. For instance, the fate of engineered AgNPs depends on the water pH and ionic strength, and constituents (e.g., natural organic matter); ionic iron species (i.e., Fe^2+^/Fe^3+^) enhance the formation of AgNPs under both thermal- and light-induced conditions and ROS play an important role in AgNP formation [81]. Noteworthily, the same complicated intra-molecular reactions and physical parameters influencing metal-NPs properties can be translated in the human body, where numerous biological important molecules and metal ions are present under different pH, oxygen, and temperature conditions.

NPs are characterized by a high surface-to-mass ratio, which enhances their chemical-physical properties with respect to parent materials. The desired industrial and medical properties of NPs can become toxic to living matter when out of control. For instance, AgNPs can easily cross the cell membrane, and in the cytoplasm inhibits mitosis, cell growth, and division [82]. Even if the bioavailability of metals in NPs can be lower than their soluble counterparts, NiNPs affect more lung epithelial cells than free nickel ions [83], probably due to NPs physical properties (i.e., their size, shape, density).

The increasing exploitation of potentially toxic NPs and NP-containing materials requires the continuous monitoring of NPs in the environment. The qualitative and quantitative analysis (in space and time) could link the NPs physical and chemical properties with their potential negative effects on living organisms, and eventually assess the effects on the development and progress of human pathologies.

Metal toxicity is well known and environmental monitoring of free metals and metal ions in the workplaces is well defined. Conversely, specific metrics of NPs exposure, and hazards to workers, are limited, while the precise mechanism of NPs toxicity is unknown. Nowadays, the monitoring of nanoparticles includes the measurements of particle number concentration, mobility due to particle size distribution (in the air), particle surface area, surface topography and morphology, and nanoparticle elemental composition [84]. Importantly, NPs have different hazards and risks of exposure in different areas of the same environment [5,85,86,87,88,89,90]. For instance, NPs in the aquatic environment have potentially long-lasting adverse effects on human and ecological health [91,92,93,94,95,96], thus occupational exposure limits (OELs) are defined specifically, rather than in laboratory studies with standardized materials [97]. Nowadays, there are no OELs specific to nanomaterials.

Regarding the manufacturer NPs, the European Union regulatory framework REACH (Registration, Evaluation, and Authorization of Chemicals) defined standard regulatory toxicology tests, quantitative structure–activity relationships (QSARs), and physiologically based pharmacokinetic models (PBPKs), which should be performed before product release on market. Such data allow REACH to stipulate developing derived no-effect level (DNEL) of exposure and compare the DNEL with exposure levels from different scenarios in relevant exposure assessments [98].

### 3.2. Metal Accumulation Sites/Tissues

Respiratory, dermal, gastrointestinal, circulatory, immunological, and neurological are the main tracts for both metal ions and their NP forms [7,99]. The gastrointestinal barrier is ineffective for particles smaller than 20 μm and absorbed NPs can be further translocated via the lymphatic and circulatory system to the other organs, including the central nervous system (CNS) [100]. Moreover, metal-NPs can be used as vehicles through the axons of olfactory neurons from the nasal epithelium to the olfactory bulb of the brain [101]. The exact order of the metal-NPs translocation from the circulatory system to organs (liver, heart, spleen, bladder, kidney, and bone marrow) is unknown. The dermis has a rich supply of blood and macrophages, lymph vessels, dendritic cells, and nerve endings [102], thus metal ions and metal-NPs that cross through the stratum corneum and into the epidermis can be taken up the macrophages that may further translocate them to other tissues.

Metal-NPs exhibit their negative effects due to the toxicity of metal ions from which they are made—their shape or their small size. Metal-NPs can be formed in vitro and then enter a human body, or in situ due to metal precipitation and/or aggregation, often in the presence of biomolecules and drugs. In vitro, engineered metal-NPs differ from environmental metal-NPs by the regular shape. Nevertheless, engineered metal-NPs, which enter the human body, can be significantly altered by the biochemical processes, particularly during the translocation process, and the morphological analysis is inefficient to establish the etiology of the metal deposits.

The scientific literature presents numerous in vitro cellular studies and in vivo animal studies devoted to the toxicity of metal nanoparticles. In comparison, the data related to toxicity in humans are scarce, and in most cases limited to separated case studies. In the 1990s, Gatti et al. discovered that solid, inorganic, non-biodegradable particles produced by many different sources, often inadvertently or as side effects of some industrial processes, can enter the human body without it being able to eliminate them [103,104,105] while translocating to different organs [99,106,107]. Recently, Gatti et al. presented a series of pharmacy products that accidentally contained nanoparticles and led to the development of severe pathologies documented in case studies [108].

Different case study reports present electron microscopy images of nanoparticles embedded in the organ tissues, supported by the qualitative X-ray analysis of metal content in nanoparticles. Nevertheless, it is unclear whether presented aggregates are formed in vivo due to metal accumulation, and related biochemical processes, or outside the human body. Human evolution has been assisted by the presence of different metals and their ionic form in natural habitat, so that human body developed numerous defense mechanisms against metal (both essential and toxic) overload (e.g., synthesis of peptides and proteins chelating metal ions (glutathione); translocation of toxic metal excess into bones). Indeed, reference man quantitative analysis [109] of metal content in different tissues shows the presence of numerous metals, also those considered toxic, in healthy subjects.

Recently, we presented pioneering studies of metal aggregates in the kidneys and livers of healthy subjects [110]. In contrast to case studies correlating pathology, often with unknown scientific etiology, with toxicity to metal nanoparticles, we examined samples of 35 healthy subjects. Scanning electron microscopy (SEM) coupled with energy-dispersive X-ray spectroscopy (EDS) microanalysis of 513 particle aggregates (276 in the liver and 237 in the kidney samples), showed that the ubiquitous presence of metal species as particle aggregates in human tissues could be a condition of normality and is far from being used as a toxicity biomarker.

### 3.3. Drugs Enhancing/Involved in Metal Dyshomeostasis

Hypertension is widely diffused in Western societies and the prime therapy is based on antihypertensive drugs, which must be taken for the long term. Antihypertensive drugs lead to magnesium, sodium, potassium, and calcium dyshomeostasis [111]. The analysis of Brown et al. [112] showed that the use of angiotensin-converting enzyme (ACE) inhibitors and angiotensin 2 receptor antagonists or thiazide diuretics reduces zinc levels. For instance, urinary zinc increases in patients treated with captopril (from 50 mg/day), enalapril (20 mg/day), losartan (50 mg/day), losartan (50 mg/day) together with hydrochlorothiazide (12.5 mg/day), captopril (75 mg/day) together with frusemide (40 mg/day) and stand-alone hydrochlorothiazide (25 mg/day). Serum zinc content also decreases with captopril (50–150 mg/day), verapamil (240 mg/day), atenolol (50–150 mg/day), and the combination of losartan (50 mg/day) and hydrochlorothiazide (12.5 mg/day). All listed drugs have different metal-binding sites and form stable complexes not only with zinc but also with copper ions [113]. The possibility that long-term use of antihypertensive drugs affects zinc and copper homeostasis and pharmacokinetics must be taken into consideration in the treatment of patients with conditions correlated with the unbalance of zinc homeostasis, such as diabetes, liver cirrhosis, bowel diseases, and impaired immune function [114].

The scientific literature shows numerous pairs of drug-metal complex formation and drug–metal competition interactions. For instance, penicillamine reduces significantly absorption of dietary zinc [115], while the dietary zinc can interact with different drugs and reduce their absorption and/or effectiveness, among which cinoxacin, ciprofloxacin, enoxacin, gatifloxacin, grepafloxacin, levofloxacin, moxifloxacin, norfloxacin, ofloxacin, tetracycline, gemifloxacin, sparfloxacin [115]. Other dietary metals that reduce the bioavailability and/or effectiveness of drugs are magnesium and iron [115]. A number of drugs have metal-binding sites that form iron-drug (as well as other essential metal ions) complexes.

Drug functional chemical groups, which bind metal ions, contain mainly oxygen, nitrogen, and/or sulphur [116]. These functional groups (e.g., phenolic, catechol, carboxyl, amine, and sulphydryl) have electron pairs that chelate metal ions [116]. Functional groups are weak acids or bases and thus the metal binding depends on pH conditions. The strength and stability of the metal/drug complex are described by the stability constant, which can be predicted by analyzing the drug structure. Metal-drug complexes with high stability constants are more likely to form metal-drug adducts. The increased or decreased solubility (e.g., due to changing net charge) of the formed metal-drug complexes in aqueous solutions (e.g., gastrointestinal fluids) is another condition, which may lead to adverse effects. Precipitation of the metal-drug complex not only decreases the availability of both but may also lead to the formation of insoluble aggregates that cause inflammation and lead to the development of pathological states. On the contrary, increased solubility of the metal-drug complex may change the known route of drug biodistribution.

Lithium is a naturally abundant metal, found in groundwater, and the concentration of lithium circulating in the healthy human is approximately 2.5 lEq/L (roughly 1/500th of the plasma level of a patient in lithium therapy [117]). Noteworthily, there is a hypothetic correlation between the higher lithium content in tap water and the decreased incidence of violent behavior in society (less suicidality, homicidality, and rape) [118,119,120]. Multiple case reports and observational data showed that diuretic (thiazide, loop, potassium-sparing, osmotic, and methyl xanthine diuretics) and antihypertensive (i.e., ACE inhibitors and angiotensin II receptor antagonists) drugs interact with lithium. Moreover, different non-steroidal anti-inflammatory drugs significantly lower the mean change of lithium clearance and lead to lithium intoxication. An exhaustive review about lithium-drug interaction has been presented by Finley [121].

Recent studies showed that free copper ions—as well as their copper-drug complexes (e.g., with metformin)—interact with DNA, leading to mutations and errors during transcription, and unwinding and/or rewinding of the double helix [122]. Moreover, copper redox potential is biologically significant and thus can cause oxidative DNA damage and strand breaks [123]. The toxic mechanisms of copper-drug complexes can be effectively used in cancer therapy but must be taken into consideration in drug therapies in the pathologies correlated with copper unbalance, e.g., diabetes. For instance, metformin is the first-line treatment in type 2 diabetes mellitus (T2DM), while Copper(II)/metformin complexes have high stability and can bind to biologically important molecules (e.g., DNA and GSH) and have shown promising results in vitro as anti-cancer therapies [124].

The list of drugs, often taken life-long in the therapy, that form stable complexes is long; nevertheless, little is known about the clinical effects. Further studies are required to establish the clinical significance of chemical interactions between metal ions and drug molecules, and the possible role in metal dyshomeostasis in the body.

### 3.4. Diagnosis

Metal intoxication pathologies are hard to diagnose, while there are no common and characteristic symptoms. The harmful effects of metal ions depend on metal type, exposure dose and duration, presence of enhancing factors, and the individual response of defense mechanisms. In clinical praxis, patients present various symptoms ranging from oral mucosal changes and skin pathologies to excessive fatigue and autoimmune diseases.

In vivo metal ions activate T-cells, initiating systemic inflammation, which, through cytokines, affects the cells and adjacent tissues [125]. For instance, metal ions released from dental prostheses or implants may also affect the brain and hypothalamus–pituitary–adrenal (HPA) axis.

The most common clinical reactions to metals are local skin reactions or systemic reactions (fever, profound fatigue, multiple chemical sensitivity), but are not expressed by all exposed individuals. Separated case-control studies of single patients or with few participants, who have often different genotypes, are of limited value [126].

Metal intoxication can be confirmed by the qualitative and quantitative analysis of metal content. In clinical practice, the choice of biological samples for metal analysis must consider the sites of metal accumulation and/or routes of translocation. The analysis of biological liquids (e.g., blood, urine, saliva) are non-invasive, fast, and economic; nevertheless, their metal content lowers, when the time after exposition increases, and few metals are an exception. Prolonged metal exposition, often at low doses, requires the analysis of tissues, where the toxic metal accumulates and leads to the development of a pathological state. The analytical methods used in metal analysis vary and depend on the type of samples under analysis.

The liver metabolizes drugs and toxins and is also the main metal storage tissue when the metal intoxication occurs. The liver biopsy for metal analysis provides valuable data in the recognition of metal-induced pathology. Inductively coupled plasma (ICP) qualitative and quantitative analysis of metal content in biopsy samples in combination with SEM with energy dispersive X-ray analysis (SEM-EDX) are executed in clinical practice when non-invasive markers suggest elevated metal concentrations. Nevertheless, SEM-EDX is often not sensitive enough for trace element imaging. Moreover, the heterogenous distribution of metals in the liver can result in inaccurate analytical data. MRI allows the mapping only of selected single metals in organs [127]; for instance hip magnetic resonance in metal artifact reduction sequence (MARS): periprosthetic materials with intermedium T1 signal and T2 hyperintensity were compatible with an adverse reaction to metal debris [128]. Nevertheless, the number of metals detectable with MRI is reduced and the quantitative analysis is often difficult. Therefore, other imaging techniques, which analyze the regional distribution of individual or group of metals, combined with a diagnostic score system are needed [129].

Laser ablation inductively coupled plasma mass spectrometry (LA-ICP-MS) was proposed by Pornwilard et al. [129] in clinical practice for identification and evaluation of hepatic metal disorders and to detect metal (iron, manganese, zinc, copper, and cadmium) variations during ongoing hepatic fibrogenesis. The technique was also efficient in the analysis of other essential and toxic metal ions (calcium, chromium, cobalt, molybdenum, silver, tin, mercury, and lead) in human tissues. More detailed data of different diagnostic imaging techniques for hepatic metal disease are summarized in the recent review by Susnea et al. [130].

Up to now, human genes activating metal-induced inflammation and autoimmunity, as well as genetic markers of genetic susceptibility, are not yet known [125], and one must rely on the (phenotypic) biomarkers.

Biomarkers are defined as indicator-signaling events in biological systems or samples (body fluids, cells, or tissues) [131], and are used to characterize exposure, effect or susceptibility. Exposure biomarkers describe the internal dose, for instance, the quantity of the compound (its metabolites or conjugates) in biological samples such as urine, blood, hair. Biomarkers of effect measure changes in biochemical, physiological, or behavioral processes in an affected organism, e.g., altered structure and/or function of the proteins. Biomarkers of susceptibility present individual predisposition to a given compound, for instance, due to genetic polymorphisms.

Detoxification enzymes, e.g., apolipoprotein E, glutathione s transferase T1 (GSTT1), and glutathione s transferase M1 (GSTM1), are the primary biomarkers of metal harmful effects. Mutation in the gene encoding apolipoprotein E (substitution of cysteine with arginine—an amino acid lacking thiol groups) is a susceptibility biomarker for Alzheimer’s disease [18], while homozygous deletion of GSTT1 and combined deletion of GSTT1-/GSTM1- was found in patients sensitized by thimerosal (Westphal’s group) [132]. Therefore, non-invasive serum parameters (e.g., serum transferrin concentration and saturation, ferritin, hemoglobin, hematocrit, serum ceruloplasmin) and genetic testing of causative genes are widely propagated in diagnosis to identify metal disorders or predisposition for respective diseases.

In clinical practice, one of the best-known metal intoxications is caused by mercury. Blood mercury concentrations rapidly increase immediately after or during brief exposure, and in those who have been chronically exposed to mercury, blood mercury concentration levels maintained a high level even when the exposure ceased, due to the heavy burden of mercury on the body [133]. Urine concentration of metallic or inorganic mercury compounds is very stable, while organic mercury (e.g., methylmercury) is usually excreted into the feces [134]. The first laboratory tests to diagnose mercury intoxication are complete blood cell count, electrolyte assays, and renal and hepatic function tests. Nevertheless, lab tests do not evaluate the influence of mercury exposure on health. For this reason, electrocardiography, pulmonary function test, cardiovascular monitoring, electroneuromyography, and neuropsychological tests are executed [135]. Evaluating the influence of mercury exposure on health and establishing treatment standards are very difficult. To date, there are no universal diagnostic criteria for mercury overload. A review in the U.S. in 2012 suggested that when mercury is excreted in the urine at levels in the National Health and Nutrition Examination Survey (NHANES) reference range of two standard deviations or higher by a provocation test, the victim is overexposed to mercury [136]. Symptomatic patients with mercury poisoning warrant immediate treatment with chelating agents. However, it is unclear whether therapy with chelating agents is truly beneficial in severely intoxicated patients. Furthermore, indications for the therapy have not yet been fully established [137].

Cobalt intoxication has been known in the context of so-called Quebec beer drinkers’ cardiomyopathy and hard steel work-related exposure to cobalt [138,139]. Nowadays, cobalt intoxication is a rare complication, nevertheless, occurring in metal-on-metal (MoM) arthroplasty [140]. Clinical manifestations are highly heterogenous symptoms involving hematopoietic system, hypothyroidism [141], reversible cardiomyopathy [141,142], acute kidney tubular injury, peripheral neuropathy and malignancy [141,143], pseudotumor (a solid, or cystic granulomatous formation that models around the prosthesis [144]). MoM arthroplasties release metal oxides, which react and/or accumulate (as metal phosphates) within the synovia and surrounding tissues, and are further translocated by macrophages to the lymph nodes (leading to lymphadenopathies [141]). Moreover, a type IV hypersensitivity reaction with perivascular T-lymphocytes and plasma cells infiltrate leads to the occurrence of a pseudotumor surrounding the prosthesis [141,143,144]. The pseudotumor can vary in size and can be filled by solid or fluid content, the latter forming a peri-prosthetic effusion. The pseudotumor effusion may compress bones and soft tissues and leads to destructive phenomena such as aseptic fibrosis, osteolysis, soft tissue necrosis, arthroplasty mobilization, pathological fractures, and dislocations [144].

### 3.5. Therapy for Metal-Related Pathologies

Early recognition of metal intoxication permits the removal of the metal source (e.g., prosthesis) and/or an immediate reduction in the absorbed dose and supportive therapy, which helps to eliminate the absorbed dose (e.g., dialysis). Absorbed toxic metals can be inactivated using chelation or competition therapy.

Generally, chelation therapy removes toxic metal ions from vulnerable tissues. This requires a high metal affinity of the chelating drug, much higher than the affinity of biological molecules [145], which are present in high concentrations. Low molecular weight compounds such as cysteine, arginine, glutamate, citrate, and glutathione, as well as proteins [145] are first-line defense mechanisms against metal toxicity, and many toxic metal ions are present in the human body at low concentrations without leading to pathological states.

The first two drugs used in clinical chelation therapy were 2,3-dimercaptopropanol (BAL), used for intoxications of arsenic, and ethylenediaminetetraacetate (EDTA) used in the treatment of lead intoxication [146]. Nowadays, the use of BAL and EDTA is limited due to parenteral administration, their toxicity, and the side effects due to toxic metals redistribution into the brain [147]. In addition, the use of BAL leads to side effects, including sweating, fever, hypertension, headache, nausea, vomiting, and palpitations [148,149,150].

The finding of essential metal ions (mainly iron, copper, and zinc [151,152,153,154]) and some toxic metal ions (aluminum, arsenic, bismuth, cadmium, mercury, lead, thallium, and titanium [155,156]) in the plaques present in the brains of patients affected by neurodegenerative disorders (mainly AD and PD) arouse the interest of metal chelation therapy. Compounds, which chelate neurodegeneration relevant metal ions (i.e., copper(II), copper (I), iron(III), iron(II), manganese(II), and zinc(II)) are aimed to target metal dyshomeostasis and alleviate the pathological state.

A recent review by Tosato et al. [157] collected data of 800 known compounds that were used, tested, or proposed for PD therapy from 2014–2019 (April). Approximately 250 among them had theoretical or experimentally ascertained metal-chelating properties towards copper(II), copper(I), iron(III), iron(II), manganese(II), and zinc(II).

The first drug with metal-binding ability used in the therapy of AD was clioquinol [158], which unfortunately is unsuitable for long-term use due to several side effects [159]. Its successors are other metal-binding drugs and their derivatives: triazole [160,161,162,163,164,165] deferiprone [166,167,168], 8-hydroxyquinoline [158,169,170,171,172,173], cyclam [174,175,176,177,178], thioflavin T [179,180,181,182,183], p-I-stilbene [184,185], chalcone [163,164], resveratrol [186,187], flavone [188,189], donepezil [190,191], tacrine [192,193], and dopamine [194]. Nevertheless, chelating agents that remove metal overload from the cell have proven to be only partially effective in the treatment of neurodegeneration [195].

Metal-chelating drugs form stable complexes with metal ions, but mostly lack metal-specificity and can remove also essential metal ions leading to side effects and toxicity [196]. Moreover, peptides and proteins involved in mechanisms of essential metal ions homeostasis, lack metal selectivity when the concentration of competing metal ions is abnormally high. Such a ‘biological bug’ can be efficiently used in therapy. For instance, zinc is a natural competitor of copper, and zinc therapy in Wilson’s Disease is as effective as D-penicillamine treatment [197]. Zinc supplementation has a considerable influence when cadmium exposure is moderate or relatively high, as it reduces the toxic effects of cadmium in the body [198].

Metal protein attenuating compounds (MPACs) are a novel class of drugs that compete with the target protein for metal ions, and in the consequence reduce the protein capacity to sequester metal ions [199]. This concept should not be confused by the concept of “chelating agents” mentioned above, which removes bulk metals, e.g., copper excess in Wilson’s disease [199]. The first compound used as an MPAC was the previously mentioned clioquinol [200], which restores metal homeostasis and decreases the quantity of Aβ peptides [201].

## 4. Conclusions

Human life depends on the proper balance of 10 essential metal ions, which play structural and regulatory roles in the body. The unbalance of essential metal ions and intrusion of other metal ions (considered toxic) lead to the development of pathological states. There are no characteristic symptoms of metal intoxication, hence the proper diagnosis is difficult. Environmental monitoring of metal and metal-nanoparticles helps to reduce hazards and identify metal-related pathologies when intoxication occurs. Biomarker and metal qualitative and quantitative analysis are of paramount importance in the choice of proper therapy in the treatment of diseases caused by metals and metal-nanoparticles. Importantly, metals used as drugs, as well as drugs with metal-chelating ability, may lead to metal dyshomeostasis, and patients under therapy should be screened for metals concentrations.

## Figures and Tables

**Table 1:** Causes and pharmacological therapy in metal-related genetic disorders.

Metal	Disease	Gene	Affected Tissue	Therapy
Mn	Manganese transporter deficiency	SLC30A10 SLC39A14 (manganese transporter)	Liver, Nervous system	Manganese(II)-sulfate monohydrate [10]
Fe	Aceruloplasminemia	CPL (Ceruloplasmin) CP (Ferroxidase)	Liver, pancreas, nervous system	Iron chelation (Deferoxamine, Deferasirox) + Vitamin E and C/+ Fresh Frozen Plasma, Zinc administration, Minocycline administration, Enzyme Replacement Therapy, Gene Therapy [11]
	Neuroferritinopathy, Hyperferritinemia-cataract syndrome, L-ferritin deficiency	FTL (iron storage)	Nervous system	Iron chelation (Deferoxamine, Deferasirox), dopamine-related drugs [12]
	Spastic paraplegia type 35	FA2H (fatty acid 2-hydroxylase (Synthesis of sphingolipids))		Botulinum toxin injections, microtubule destabilizing drugs (e.g., vinblastine) [13]
	HARP syndrome (hypoprebetalipoproteinemia, acanthocytosis, retinitis pigmentosa, and pallidal degeneration)	PANK2 (Pantothenate kinase (CoA synthesis))		No therapy
	Pontocerebellar hypoplasia type 12	COASY (CoA synthesis)		
	Infantile neuroaxonal dystrophy 1, Neurodegeneration with brain iron accumulation 2B, Parkinson’s disease type 14	PLA2G6 (Phospholipase)		
	Spastic paraplegia 43, Neurodegeneration with brain iron accumulation 4	C19orf12 (Mitochondrial magnesium homeostasis)		Intrathecal baclofen [14]
	Woodhouse–Sakati syndrome	DCAF17 (Ubiquitinylation)		Treatment is symptomatic (e.g., hormone replacement therapy for hypogonadism) [15]
	Neurodegeneration with brain iron accumulation type 5	WDR45 (Autophagy)		Treatment is symptomatic [16]
	Kufor–Rakeb syndrome, Spastic paraplegia type 78	ATP13A2 (Lysosomal divalent cation (transition metal) transporter)		Treatment is symptomatic [16]
	Hereditary hemochromatosis	HFE1 (HFE protein), HJV (Hemojuvelin), TrR2 (Trasferrin receptor-2), SLC40A1 (Ferroportin), HAMP (Hepcidin)	Liver, pancreas, heart	Therapeutic phlebotomy, iron chelating therapy, erythropoietin [17]
Cu	Wilson’s disease	ATP7B (beta polypeptide, ATPase, Cu^II^ transporting)	Liver, brain, kidneys, cornea	Copper chelation (e.g., Penicillamine, Trientine), zinc supplementation, Tetrathiomolybdate [18]
	MEDNIK syndrome	AP1S1 (adaptor protein complex 1 subunit β1)	liver, nervous system	Zinc supplementation (e.g., zinc acetate) [19]
	Menkes Disease	ATP7A (ATPase Copper Transporting Alpha)	Nervous system, skeletal, skin	Copper supplementation (e.g., copper histidine) [20]
	Occipital Horn Syndrome (OHS)	ATP7A (P-type ATPase)	Nervous system, skeletal, skin	Copper supplementation, disulfiram [21]
	Huppke-Brendel Syndrome (HBS)	SLC33A1	Nervous system	Treatment is symptomatic [22]
Zn	Acrodermatitis Enteropathica	SLC39A4 (Solute Carrier Family 39 Member 4)	Liver	Zinc supplementation [23]
	Transient Neonatal Zinc Deficiency	SLC30A2 (Solute carrier family 30 member 2)	Skin	Zinc replacement therapy [24]
	Ehlers-Danlos Syndrome	SLC39A13 (zinc transporter ZIP13)	Nervous system, Muscle, skeletal	Nutritional supplements [25]
	Birk-Landau-Perez Syndrome	SLC30A9 (Zinc transporter 9)	Nervous system, kidneys,	Symptomatic Therapy [26]
Se	Keshan Disease	Under investigation, genes related to selenoproteins and thioredoxin reductase	Heart	Selenium supplementation [27]
	Rigid spine muscular dystrophy 1 (RSMD1) and congenital myopathy with fiber-type disproportion	SEPN1 (Selenoprotein N)	Muscle nervous system	No approved drug therapies [28]

## Data Availability

The data presented in this study are available on request from the corresponding author.

## Abbreviations

2,3-dimercaptopropanol (BAL); Alzheimer’s Disease (AD); amyloid beta (Aβ); angiotensin converting enzyme (ACE); apolipoprotein E (APOE); central nervous system (CNS); copper nanoparticles (CuNPs); copper oxide nanoparticles (CuO NPs); derived no-effect level (DNEL); electrocardiogram (ECG); energy dispersive X-ray (EDX); energy dispersive X-ray spectroscopy (EDS); ethylenediaminetetraacetate (EDTA); glutathione (GSH); glutathione peroxidase 4 (GPX4); glutathiones transferase M1 (GSTM1); glutathione s transferase T1 (GSTT1); gold nanoparticles (AuNPs); Huppke-Brendel Syndrome (HBS); Inductively coupled plasma (ICP); Laser ablation inductively coupled plasma mass spectrometry (LA-ICP-MS); Magnetic Resonance Imagining (MRI); Mesoamerican Nephropathy (MeN); metal artefact reduction sequence (MARS); Metal protein attenuating Compounds (MPACs); metal-on-metal (MoM); nanomaterials (NMs); Nanoparticles (NPs); National Health and Nutrition Examination Survey (NHANES); Occipital Horn Syndrome (OHS); palladium nanoparticles (PdNPs); Parkinson’s disease (PD); positron emission tomography (PET); progressive supranuclear palsy (PSP); quantitative structure activity relationships (QSARs); reactive oxygen species (ROS); Registration, Evaluation, and Authorisation of Chemicals (REACH); Rigid Spine Muscular Dystrophy 1 (RSMD1); Scanning electron microscopy (SEM); Silver nanoparticles (AgNPs); superoxide dismutase 1 (SOD1); titanium dioxide nanoparticles (TiO_2_ NPs); Type 2 Diabetes Mellitus (T2DM); zinc oxide nanoparticles (ZnO NPs).
